# Poor overall survival in hyperhaploid multiple myeloma is defined by double-hit bi-allelic inactivation of *TP53*

**DOI:** 10.18632/oncotarget.26589

**Published:** 2019-01-22

**Authors:** Cody Ashby, Ruslana G. Tytarenko, Yan Wang, Niels Weinhold, Sarah K. Johnson, Michael Bauer, Christopher P. Wardell, Carolina Schinke, Sharmilan Thanendrarajan, Mauricio Zangari, Frits van Rhee, Faith E. Davies, Jeffrey R. Sawyer, Gareth J. Morgan, Brian A. Walker

**Affiliations:** ^1^ Myeloma Center, University of Arkansas for Medical Sciences, Little Rock, AR, USA; ^2^ Department of Pathology, University of Arkansas for Medical Sciences, Little Rock, AR, USA

**Keywords:** myeloma, double-hit, bi-allelic, TP53, survival

## Abstract

Hyperhaploid multiple myeloma is a rare numerical aberration group defined by a range of 24-34 chromosomes, which is associated with a poor prognosis with a 5-year survival rate of 23%. Hyperhaploid patient samples (n=8) were sequenced and copy number and mutations identified. Samples had a median of 13 monosomies (range 12-14), which in general were those not associated with trisomies in hyperdiploid samples. The chromosomes traditionally trisomic in hyperdiploid myeloma were disomic in hyperhaploid myeloma with retention of heterodisomy. We examined the hyperhaploid samples for frequently mutated genes and found that 8/8 (100%) hyperhaploid samples had a mutation in *TP53*, exceeding the overall rate of mutation in newly diagnosed patients (5.5%), indicating an oncogenic dependency in this group. All samples with *TP53* mutation also had monosomy of chromosome 17, indicating bi-allelic inactivation of *TP53*. As such, this high risk group is part of double-hit myeloma.

## INTRODUCTION

Multiple myeloma (MM) is a plasma cell disorder characterized by multiple complex numerical and structural abnormalities [1]. Hyperdiploidy (47-57 chromosomes) is found in 50-60% of patients and is characterized by gains of whole chromosomes, consisting mostly of odd numbered chromosomes including 3, 5, 7, 9, 11, 15, 19, and 21. The remainder of patients have translocations involving the *IGH* locus on chromosome 14 and are associated with a hypodiploid karyotype and a poor prognosis.

Hyperhaploidy is defined as a karyotype with 24-34 chromosomes and is rare in MM [2, 3], but is seen in many cancer types, including acute lymphoblastic leukemia (ALL), myeloid leukemias, chondrosarcomas, and squamous cell carcinomas [4–6]. Hyperhaploidy in MM occurs with monosomy of many chromosomes, but those chromosomes associated with trisomies in hyperdiploidy remain disomic. In ALL, a different set of chromosomes retain disomy, namely 14, 18, and 21. Interestingly, in all cancers with hyperhaploidy, including MM, chromosome 18 is usually disomic, suggesting that disomy of chromosome 18 is required for a viable cell.

We have previously shown that this group of patients is associated with a poor prognosis, with a 5-year survival rate of only 23% [2], and this was assumed to be linked to deletions of 17p and amplification of 1q in these samples. However, no sequencing of hyperhaploid samples has been performed to determine if there is a mutational profile associated with this high risk group. Recent studies have shown that del17p is not solely responsible for the poor prognosis associated with MM, and that mutations in the tumor suppressor gene *TP53* are more prognostic [7–9]. However, when mutations and deletions are taken into account there is a profound effect on prognosis when both alleles are affected, resulting in biallelic inactivation. We have recently shown that biallelic inactivation of *TP53* is a feature of Double-Hit MM, defining 6.1% of newly diagnosed MM patients with a median progression free survival of 15.4 months [10].

Herein, we have performed sequencing on a set of hyperhaploid MM samples to determine the mutational background of this rare subgroup and the mechanism in which it is generated.

## RESULTS

### Copy number profiling reveals retention of heterodisomy in hyperhaploid samples

**Figure 1:**
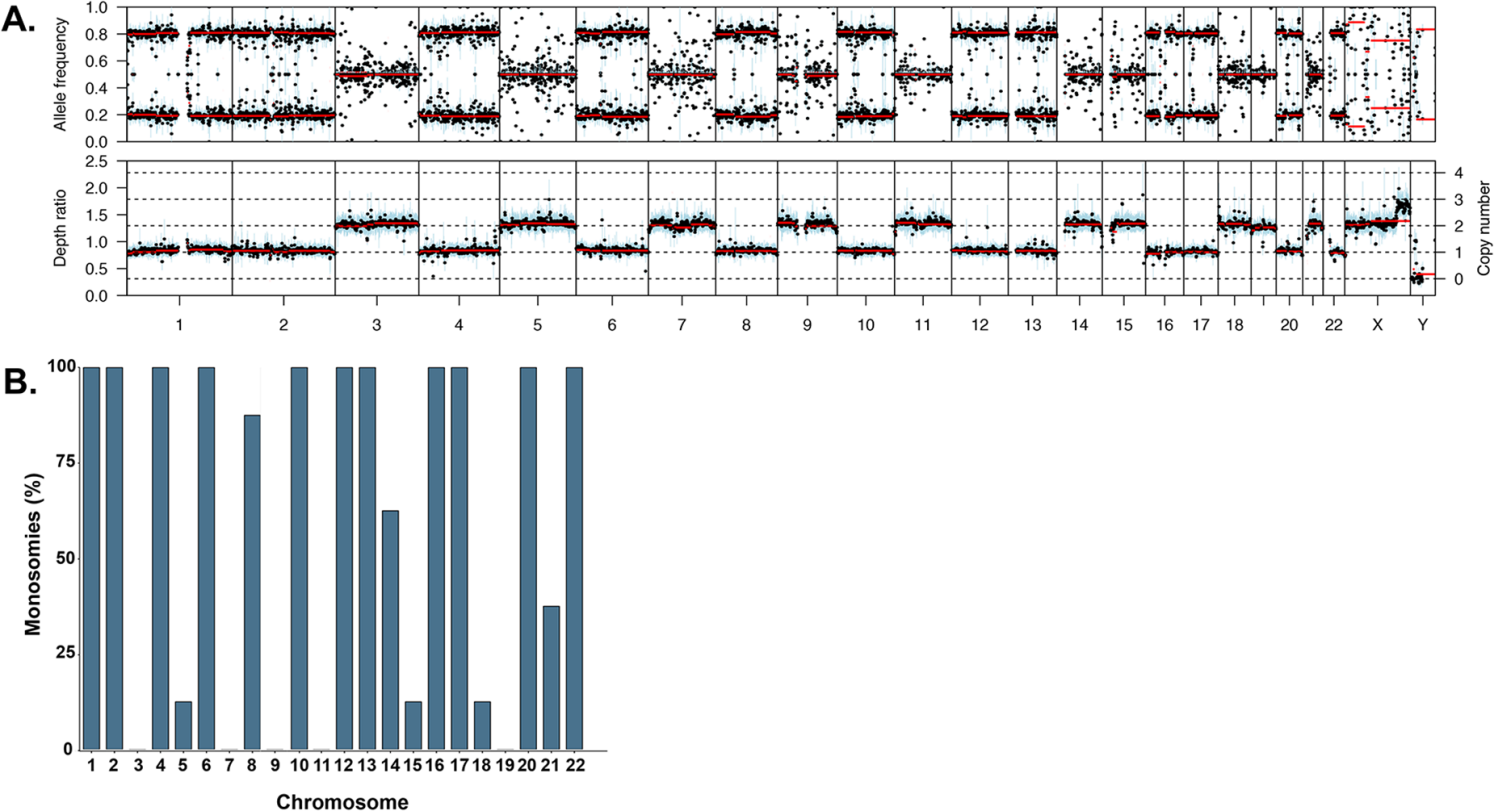
**(A)** Example B allele frequency plot showing retention of heterodisomy in the diploid chromosomes (upper) and the corresponding copy number plot (lower). **(B)** Percentage of monosomies per chromosome in the hyperhaploid samples (n=8).

We performed exome sequencing on 5 patient samples who had been identified as hyperhaploid by karyotyping and identified one additional patient sample during routine targeted sequencing. Two additional samples were identified in the MGP study dataset, giving a total of 8 hyperhaploid samples. The data were analyzed for copy number and B allele frequency. Although different methodologies were used to identify these hyperhaploid samples we do not believe that it affects the results, and that it is better to use all datasets to characterize this small subset of MM patients. Hyperhaploidy was differentiated from hyperdiploidy in exome data using B allele frequency in the tumor compared to matched normal sample. Where consistent loss of heterozygosity (LOH) was seen the ploidy of the samples was re-normalized so that those chromosomes with LOH had a copy number of one, Figure 1A.

The number of monosomies per sample varied from 12 to 14 and was more frequent on the chromosomes not associated with trisomies in hyperdiploid samples, namely chromosomes 1, 2, 4, 6, 10, 12, 13, 16, 17, 20, and 22, Figure 1B. Those chromosomes that are associated with trisomies in hyperdiploid myeloma were less likely to be monosomic, with the exception of chromosome 18. Chromosome 18 is not associated with hyperdiploidy but was disomic in 7/8 samples.

### Hyperhaploidy is generated through sequential loss of chromosomes

**Figure 2:**
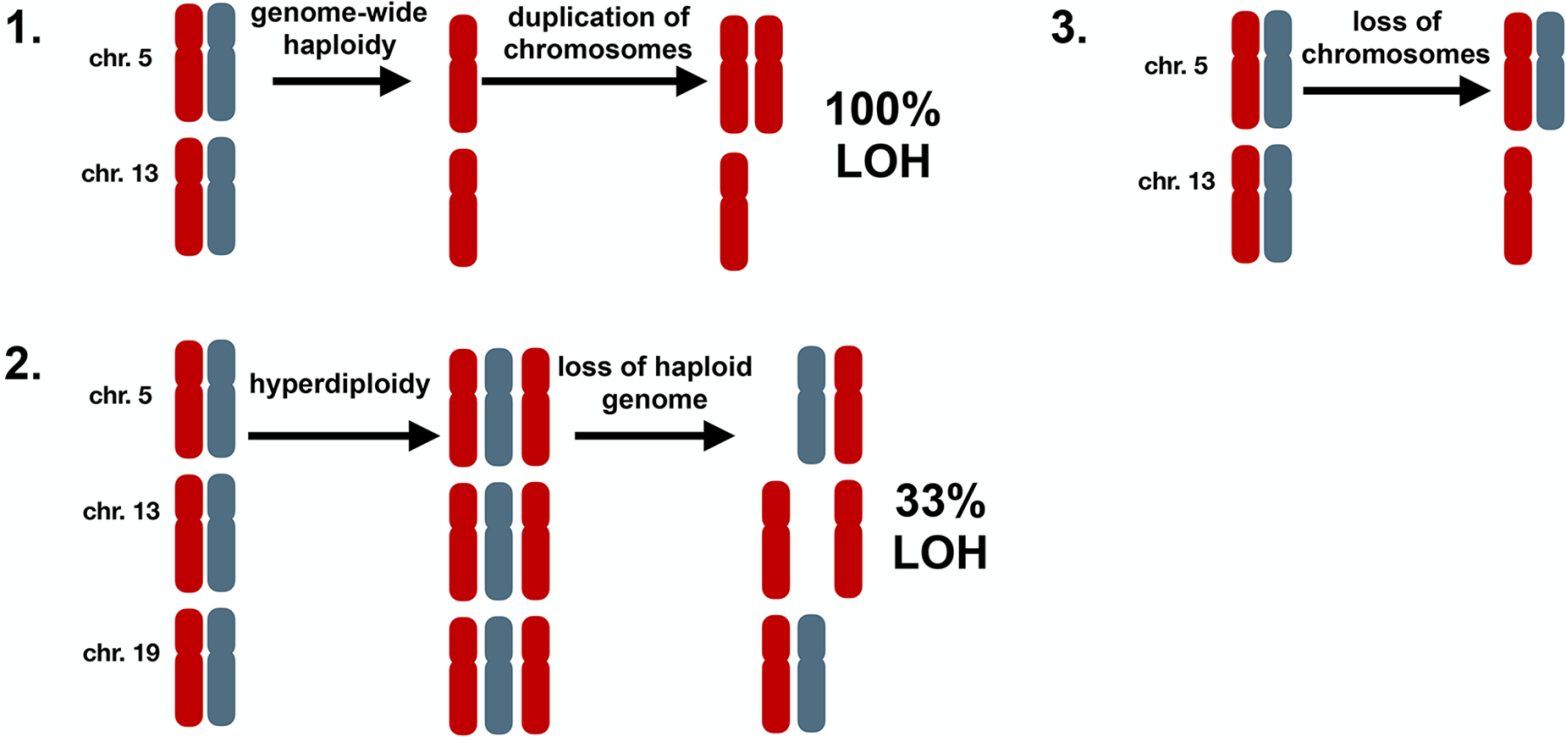
Possible mechanisms of generating hyperhaploidy 1. Cells undergo genome-wide loss of chromosomes resulting in haploidy (n=1) followed by subsequent duplication of some chromosomes (n=1.4), resulting in loss of heterozygosity (LOH) on all chromosomes. 2. Cells first become hyperdiploid (n=2.4) before losing a haploid genome (n=1.4). In this instance paternal or maternal chromosomes are lost randomly resulting in diploid chromosomes with LOH 33% of the time. 3. Cells undergo loss of chromosomes resulting in hyperhaploid state (n=1.4) with retention of heterodisomy in all diploid chromosomes, which is consistent with our results.

By examining all samples and chromosomes it was apparent that hyperhaploidy does not occur randomly. There are three possible ways in which hyperhaploidy can be generated, Figure 2: 1) Halving of a diploid genome followed by gain of odd numbered chromosomes, 2) Gain of odd numbered chromosomes followed by loss of a haploid genome, and 3) Loss of chromosomes from a diploid state. The first method would result in genome-wide LOH, which we do not detect. The second method would result in LOH occurring on one-third of disomic chromosomes, due to loss of the “odd” allele. We do not see any LOH in the disomic chromosomes, so this mechanism is unlikely. The third method would result in retention of heterodisomy on the disomic chromosomes in all cases and is compliant with our data where all disomic chromosomes retain heterodisomy.

**Figure 3:**
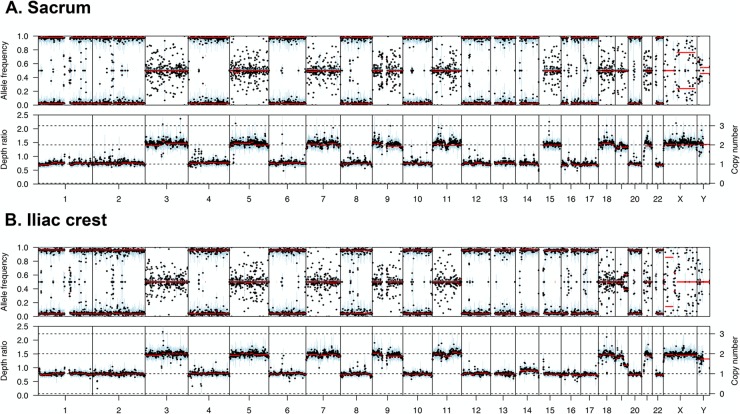
Copy number and B allele plots from two samples from the same patient **(A)** A sample taken from the left sacrum shows retention of heterodisomy of chromosome 15. **(B)** A sample taken from the iliac crest two years after the sacrum sample shows monosomy of chromosome 15, indicating gradual loss of chromosomes over time.

In support of the third hypothesis, we sequenced serial samples from one patient which were taken 2 years apart. The first sample, taken from the left sacrum, had retention of autosomes 3, 5, 7, 9, 11, 15, 18, 19, and 21. However, the second sample taken from the iliac crest two years later had monosomy of chromosome 15, Figure 3, suggesting a gradual loss of chromosomes over time or between sites in the skeleton.

### Hyperhaploidy is associated with biallelic TP53 inactivation and NF-κB pathway activation

We examined the sequencing data for mutations that may be associated with hyperhaploidy. We found that all samples had a *TP53* mutation, Table 1. The mutations were similar to those seen in other studies and were mostly clonal, with a median variant allele frequency of 0.92. Given that all the samples had monosomy of chromosome 17, the hyperhaploid samples all have biallelic inactivation of *TP53*, which is defining attribute of Double-Hit myeloma. Two of the samples had mutations in *TP53* which were not clonal, with variant allele frequencies of 0.42 and 0.59 with deletion of chromosome 17. The presence of sub-clonal *TP53* mutations with deletion of 17p suggests that deletion occurred first in these samples, followed by mutation.

**Table 1:** *TP53* mutations detected in hyperhaploid samples

Patient ID	Codon change	cDNA position	VAF
**13875**	E287fs	c.860-861insTCTTCCTCAGGTCCCCCCGGTGTAGGGA	0.92
**17188**	V73fs	c.216delC	0.96
**24138**	LGFL111-114L	c.334-342delGGCTTCTTG	0.42
**27082**	F338fs	c.1013delT	0.95
**27647**	R273H	c.818G>A	0.92
**35652**	G244D	c.731G>A	0.59
**MMRF_1364**	K292fs	c.873delG	0.96
**MMRF_1499**	F134S	c.401T>C	0.89

We examined the samples for the presence of mutations in 63 previously identified driver genes in MM [12], and found a limited number of mutations. Out of the 63 genes, mutations were found in *ATM*, *CDKN1B*, *CREBBP*, *CYLD*, *KMT2B*, *KRAS*, *MAX*, *TP53*, and *TRAF3*. Mutations were highly clonal across all driver genes, with a median cancer cell fraction of 0.91, suggesting that mutations occurred early in disease pathogenesis. Apart from *TP53*, multiple mutations were seen in *CYLD* (n=4) and *TRAF3* (n=2) implicating the NF-κB pathway as being important in hyperhaploid samples.

## DISCUSSION

Here we have performed sequencing of a set of hyperhaploid MM patient samples, which define a high risk group with a poor prognosis and a 5-year survival rate of 23%, and account for approximately 0.25% of newly diagnosed myeloma cases [2]. Other genomic markers associated with poor prognosis include deletion of 1p32, 1q12, 17p, and gain of 1q21 [13]. Hyperhaploidy results in monosomies of chromosomes 1 and 17, potentially giving reason to the poor prognosis associated with this group. However, recent studies have also shown that biallelic inactivation of *TP53*, through either mutation or deletions, is also associated with a very poor prognosis [7, 9, 10].

We have recently described Double-Hit MM to include biallelic inactivation of *TP53*, and results in a median PFS of 15.4 months [10]. We have also shown that deletion of *TP53* alone is not sufficient to result in poor outcome in several independent datasets [7, 10]. Here we have shown that hyperhaploid myeloma samples always have biallelic inactivation of *TP53*, which would explain the association with poor prognosis in this group, defining them as Double-Hit MM. The fact that the copy number abnormalities associated with hyperhaploidy include monosomy of chromosome 17 predisposes this group to a defined “first hit” upon which mutation of the remaining allele constitutes the “second hit”, driving pathogenesis and aggressive disease. This type of association between genetic markers can be described as an oncogenic dependency [12], in which the primary copy number changes prompt accrual of dependent secondary abnormalities, which in this case are mutations in *TP53*.

Hyperhaploidy is similar to hyperdiploidy in that the same set of autosomes have a higher copy number relative to the other chromosomes, with the exception of chromosome 18. Chromosome 18 retains disomy in hyperhaploid cells where we may expect monosomy based on predefined knowledge from hyperdiploid samples. The retention of chromosome 18 is seen in other hyperhaploid cancer types as well as MM, but the reason for this is unclear [4, 5, 14]. The similarity in the gain of odd numbered chromosomes between hyperhaploid and hyperdiploid states may suggest a common cell of origin, where hyperdiploid cells lose a haploid genome equivalent resulting in a hyperhaploid clone. However, previous studies have shown that no hyperdiploid clones were present in hyperhaploid samples [2]. Here we have shown retention of heterodisomy on all disomic chromosomes meaning that the hyperhaploid cells cannot have originated from a hyperdiploid cell, based on the chances of generating copy number neutral LOH. Therefore, we suggest that hyperhaploidy is generated through loss of chromosomes, which may happen as one catastrophic cell division or as successive loss of chromosomes. This method is similar to the proposed mechanism of gain of chromosomes in hyperdiploidy, in which samples may show a dominant clone where all cells have the same gains of chromosomes, or other samples may have subclones showing successive gains of chromosomes [15].

The NF-κB pathway is frequently activated in MM through inactivation of negative regulators of the pathway (e.g. CYLD, TRAF3, BIRC2, BIRC3) or over-expression of positive regulators (e.g. NIK) [16–18]. In this set of hyperhaploid MM samples we identified mutations in *CYLD* or *TRAF3* in three patients (37.5%). We have previously shown that mutations in *CYLD* and *TRAF3* are associated with non-hyperdiploid karyotypes with a t(4;14). In this respect, the hyperhaploid mutational spectra does not resemble hyperdiploidy, but has more in common with the high risk t(4;14) group, with more NF-κB mutations, and deletions of 1p, 13q, and 17p.

In conclusion, we have shown that hyperhaploid MM is a subgroup of Double-Hit MM, with biallelic inactivation of *TP53*, which results in a poor prognosis. The hyperhaploid karyotype results from loss of chromosomes, either in one catastrophic cell division or successive losses, and does not originate from a hyperdiploid clone.

## MATERIALS AND METHODS

### Patient sample selection

Samples previously identified as hyperhaploid by karyotyping were selected for whole exome sequencing. An additional hyperhaploid sample was identified through routine targeted sequencing. Samples had undergone CD138+ cell selection by either AutoMACS (Miltenyi) or RoboSep (Stem Cell Technologies) and DNA was extracted. Patient matched control DNA was also isolated from peripheral blood stem cell harvest samples.

### Exome and targeted sequencing

DNA was prepped for sequencing using previously described protocols [11]. Briefly, 100 ng of DNA was fragmented, end-repaired, and adapters ligated using the HyperPlus kit (KAPA Biosystems). After PCR amplification the libraries were hybridized with probes against either the entire exome (MedExome, Nimblegen) or a targeted panel of 140 genes using SeqCap reagents (Nimblegen). Hybridized libraries underwent further amplification before being sequenced on a NextSeq500 (Illumina).

### Sequence analysis

Targeted panel and Myeloma Genome Project (MGP) samples were analyzed in the manner described previously [12]. For MedExome samples, FastQC (v0.11.5) was used for basic quality control of Illumina paired-end sequencing data. Sequences were aligned to reference genome hg38 using BWA (v.0.7.17). Samples were de-duplicated using Picard Tools (v.1.85). Variants were called using Strelka (v2.8.3), variant annotation was provided by Variant Effect Predictor (v85), and filtered using fpfilter (https://github.com/ckandoth/variant-filter). Copy-number alterations were determined using Sequenza (v2.1.2.14). The median coverage for MedExome samples was 115x (range: 108-143x) and for the targeted panel sample was 213x. Data have been deposited at the European Genome-Phenome Archive under accession number EGAS00001003203.
